# Phylogenetic Placement of *Whittingtonocotyle* Neto, Rodrigues & Domingues, 2015 (Monopisthocotyla: Dactylogyridae) Inferred from the First Molecular Data of Both Described Species

**DOI:** 10.1007/s11686-026-01239-8

**Published:** 2026-03-09

**Authors:** Melissa Miyuki Osaki-Pereira, André Luiz Quagliatto Santos, Reinaldo José da Silva

**Affiliations:** 1 Institute of Biosciences, Department of Biodiversity and Biostatics, Parasitology Section, São Paulo State University (UNESP), Botucatu, São Paulo State Brazil; 2 Wildlife Teaching and Research Laboratory, Faculty of Veterinary Medicine, Federal University of Uberlândia (UFU), Uberlândia, Minas Gerais State Brazil

**Keywords:** Integrative taxonomy. phylogenetic relationships. erythrinid hosts. neotropical region.

## Abstract

**Purpose:**

This study provides the first molecular assessment of the two species of the genus *Whittingtonocotyle*, parasites of the erythrinid fish *Hoplerythrinus unitaeniatus*. The main objective was to evaluate the phylogenetic cohesion of *Whittingtonocotyle* and to explore its preliminary phylogenetic affinities within Dactylogyridae based on available molecular and morphological evidence.

**Methods:**

Morphological examinations were performed in parallel with analyses of partial LSU rDNA and COI mtDNA sequences. Phylogenetic reconstructions were conducted independently for each marker to assess the monophyly of *Whittingtonocotyle* and to evaluate its relationships with other dactylogyrid taxa included in the available comparative dataset.

**Results:**

Both molecular datasets consistently recovered *Whittingtonocotyle* as a strongly supported monophyletic lineage. In the phylogenetic reconstructions, *Whittingtonocotyle* was recovered in proximity to *Urocleidoides* species parasitizing erythrinid fishes, although deeper backbone relationships showed limited statistical support.

**Conclusion:**

Morphological data support the recognition of *Whittingtonocotyle* as a distinct genus, whereas molecular analyses indicate a close phylogenetic proximity to erythrinid-associated *Urocleidoides*. This partial incongruence, together with the limited resolution of deeper relationships, highlights the need for expanded taxon sampling and multilocus datasets to fully resolve the evolutionary placement of *Whittingtonocotyle* within Dactylogyridae.

**Supplementary Information:**

The online version contains supplementary material available at 10.1007/s11686-026-01239-8.

## Introduction

The family Erythrinidae comprises 21 freshwater fish species distributed across three genera and is endemic to South America [1, 2, 3]. Among them, *Hoplerythrinus unitaeniatus* (Spix & Agassiz, 1829) is widely distributed throughout the Amazon basin and in adjacent drainage systems, plays an important ecological and economic role, particularly in small-scale fisheries [1].

Monopisthocotyls reported from erythrinid fishes currently belong to the genera *Anacanthorus* Mizelle & Price, 1965, *Cosmetocleithrum* Kritsky, Thatcher & Boeger, 1986, *Gyrodactylus* von Nordmann, 1832, *Urocleidoides* Mizelle & Price, 1964, *Vancleaveus* Kritsky, Thatcher & Boeger, 1986, and *Whittingtonocotyle* Neto, Rodrigues & Domingues, 2015[4, 5, 6, 7, 8]. However, molecular data for monopisthocotyls infecting erythrinids remain limited and unevenly distributed among taxa. To date, only nine dactylogyrid species parasitizing erythrinid fishes have been genetically characterized [9, 10], restricting broader phylogenetic inferences for this host-associated assemblage.

Neto, Rodrigues and Domingues [4] proposed the genus *Whittingtonocotyle* based on two new monopisthocotyls species collected from *H. unitaeniatus* in northeastern Pará State, Brazil, with *Whittingtonocotyle caetei* Neto, Rodrigues & Domingues, 2015 as the type species. This genus was described exclusively based on morphological characters. It is characterized by a body divided into cephalic region, trunk, and haptor; a thin, smooth tegument; poorly developed or absent ventral cephalic lobes; two pairs of eyes with elongate granules; and a muscular, glandular pharynx with a short oesophagus. The intestinal caeca are paired, confluent posteriorly, and lack diverticula. The copulatory complex comprises a sclerotized, clockwise spiral male copulatory organ and a non-articulated accessory piece. The vagina is single, dextro-dorsal and marginal, with a heavily sclerotized sigmoid to coiled canal. The haptor bears 14 hooks, paired ventral and dorsal anchors without well-defined roots, and ventral and dorsal bars, the dorsal with an anteromedial process. Species of *Whittingtonocotyle* parasitize the gills of erythrinid fishes.

Although this genus has been clearly delimited based on morphological evidence, its evolutionary relationships within Dactylogyridae remain poorly understood. A comprehensive phylogenetic framework for Dactylogyridae was proposed by Kmentová *et al*. [11]., however, *Whittingtonocotyle* could not be included in their analyses due to the absence of available DNA sequence data, and its molecular phylogenetic position therefore remained unresolved.

In this study, we provide the first molecular data for *Whittingtonocotyle* by sequencing partial LSU rDNA and COI mtDNA from its two described species. The LSU rDNA marker has been widely used to infer phylogenetic relationships at higher taxonomic levels within Dactylogyridae, whereas COI mtDNA has been primarily applied to species delimitation and the assessment of genetic divergence among closely related taxa. Despite recent advances in the molecular phylogeny of Dactylogyridae, relationships among several lineages remain poorly resolved, particularly for genera parasitizing Neotropical freshwater fishes [12, 13, 14].

Within this context, the present study aims to provide an initial molecular placement of *Whittingtonocotyle* within the available phylogenetic framework of Dactylogyridae, evaluate its relative position among erythrinid-associated dactylogyrids, and integrate molecular and morphological evidence to improve our understanding of host associations and lineage diversity among South American freshwater monopisthocotyls.

## Materials and Methods

### Host and Parasite Sampling

In March 2025, five individuals of *H. unitaeniatus* were obtained from the Pindaíba River, located in the municipality of Cocalinho, Mato Grosso state, Brazil (14°31’40.1"S 51°41’35.0"W), through fishing using a cast net. Host specimens were identified to species level based on morphological characters. The sampling was carried out under authorization from the Brazilian Institute of Environment and Renewable Natural Resources (IBAMA; SISBIO license #60640-1). Fish were euthanized by severing the spinal cord and examined for parasites under a stereomicroscope. All experimental procedures were performed in accordance with the ethical standards established by the Ethics Committee on Animal Use (CEUA) of the University Center of Vale do Araguaia, Barra do Garças, Mato Grosso, Brazil (CEUA protocol #010/2025). Monopisthocotyls parasites were isolated from the gills; specimens intended for morphological characterization were fixed in formalin preheated to approximately 60–70 °C, whereas those designated for molecular analyses were preserved in absolute ethanol. For the examination of sclerotized structures, selected specimens were mounted in Hoyer’s or Gray & Wess medium [15]. Prevalence and mean intensity were determined following the methods proposed by Bush et al. [16].

Morphometric analyses were performed following Neto, Rodrigues and Domingues [4], using a computerized microscope equipped with image capture and analysis software under differential interference contrast (DIC) and phase contrast (LAS V3; Leica Application Suite V3; Leica Microsystems, Wetzlar, Germany).Voucher specimens were deposited in the Coleção Helmintológica do Instituto de Biociências (CHIBB), UNESP, Botucatu, São Paulo State, Brazil. Additionally, all material was registered in compliance with Brazilian legislation in the National System for the Management of Genetic Heritage and Associated Traditional Knowledge (SISGEN) under registration code #A561AF5.

### Molecular and Phylogenetic Analyses

For molecular analyses, preliminary examinations of the material were conducted to assess morphology and identify the parasite species present in the samples. Representative individuals of the target species were selected, and the posterior region of the body was used for DNA extraction, whereas the anterior portion, which contains the male copulatory complex as a diagnostic structure, was mounted in Hoyer’s medium on permanent slides as a hologenophore *sensu* Pleijel *et al*. [17]. These slides will be deposited in the CHIBB collection, along with the remainder of the mounted specimens.

Total genomic DNA was extracted from the specimens using the DNeasy^®^ Blood and Tissue Kit (Qiagen), following the manufacturer’s instructions. Fragments of LSU rDNA and COI mtDNA were amplified by conventional PCR.

For LSU rDNA, amplification was carried out with primers 382 F (5′–AGC TGG TGG AGT CAA GCT TC–3′) and 1289R (5′–TGC TCA CGT TTG ACG ATC GA–3′) [18] using the profile: 95 °C for 5 min; 40 cycles of 95 °C for 30 s, 56 °C for 30 s, and 72 °C for 2 min; followed by 72 °C for 10 min. For COI mtDNA, primers COI Mono 5 (5′–TAA TWG GTG GKT TTG GTA A–3′) and COI Mono 3 (5′–TAA TGC ATM GGA AAA AAA CA–3′) [19] were used with the profile: 94 °C for 3 min; 40 cycles of 94 °C for 30 s, 47 °C for 30 s, and 72 °C for 1 min 30 s; final extension at 72 °C for 7 min.

PCR products (2 µL) were visualized on 1% agarose gels stained with GelRed™. Positive amplicons were purified using Agencourt AMPure XP magnetic beads (Beckman Coulter, Inc.) and sequenced bidirectionally using the same primers employed for PCR amplification, with the BigDye v.3.1 Terminator Cycle Sequencing Kit on an ABI3730xl Genetic Analyzer (Applied Biosystems). Raw reads were assembled and edited in Sequencher v.5.2 (Gene Codes).

The newly obtained LSU rDNA and COI mtDNA sequences were aligned with available Dactylogyridae sequences from GenBank using MUSCLE implemented in Geneious v.7.1.3 [20], with trimming of terminal regions to remove ambiguous positions. For the LSU rDNA phylogenetic reconstruction, 73 sequences of taxonomically related species were retrieved from GenBank, and together with the newly generated sequences, a total of 76 Dactylogyridae sequences were aligned (Supplementary Information 1), resulting in a final alignment of 842 base pairs. Sequences of *Murraytrema pricei* Bychowsky & Nagibina, 1977, *Pseudorhabdosynochus epinepheli* (Yamaguti, 1938) Kritsky & Beverley-Burton, 1986, and *Pseudorhabdosynochus lantauensis* (Beverley-Burton & Suriano, 1981) Kritsky & Beverley-Burton, 1986 were used as outgroups.

For the COI mtDNA dataset, 29 sequences were obtained from GenBank and combined with the newly generated sequences, resulting in a total of 31 aligned sequences (Supplementary Information 1). After trimming ambiguous regions, the final alignment comprised 496 base pairs. The sequence of *Acanthocotyle gurgesiella* Ñacari, Sepúlveda, Escribano & Oliva, 2017 was used as the outgroup. Phylogenetic analyses were conducted separately for each molecular marker.

The best-fit evolutionary model was selected with jModelTest 2 [21] through the CIPRES Science Gateway [22] based on the Akaike Information Criterion (AIC), resulting in the GTR + I + G model for both markers. Phylogenetic inference was conducted separately for each molecular marker using Maximum Likelihood in RAxML v.8 [23] and Bayesian Inference in MrBayes [24] on CIPRES. Bayesian analyses were run for 1 × 10⁶ generations with sampling frequency set to every 100 generations (samplefreq = 100), and the first 25% of sampled trees (corresponding to 250,000 generations) were discarded as burn-in. Convergence was assessed using the standard diagnostics implemented in MrBayes, including monitoring the stabilization of log-likelihood values and the average standard deviation of split frequencies (ASDSF) between runs, which indicated adequate convergence. Nodes with posterior probabilities (pp) ≥ 0.95 were considered strongly supported. ML node support was estimated using 1,000 bootstrap replicates, and values ≥ 70% were considered robust. For the COI mtDNA dataset, all three codon positions were included in the analysis and treated as a single partition. Genetic distances were calculated under the Tamura–Nei model using MEGA12 [25], with rate heterogeneity modeled by a gamma distribution.

Phylogenetic trees were visualized in FigTree v.1.4.4 [26] and edited with CorelDraw X8.

## Results

### Morphological Analyses

**Fig. 1:**
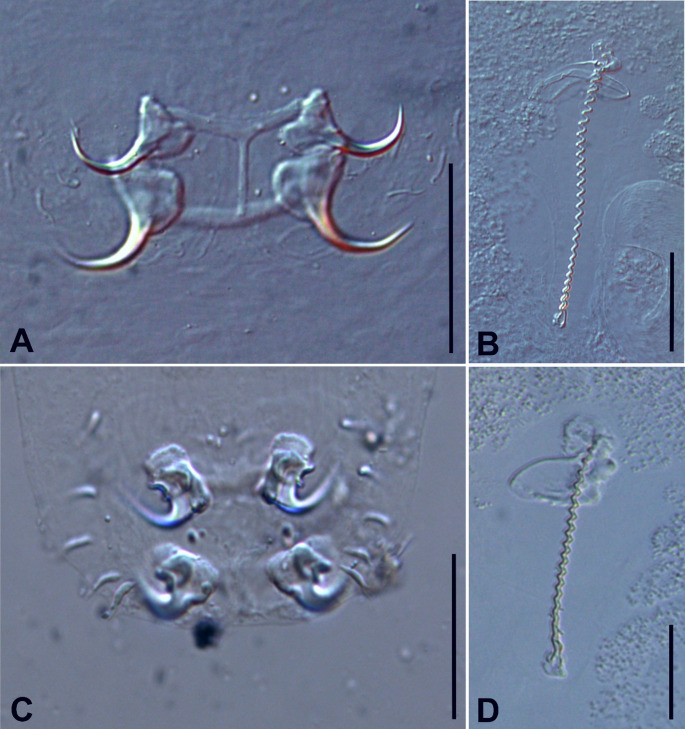
*Whittingtonocotyle caetei* Neto, Rodrigues & Domingues, 2015 (**a** Haptor and **b** Copulatory complex) and *Whittingtonocotyle jeju* Neto, Rodrigues & Domingues, 2015 (**c** Haptor and **d** Copulatory complex) found parasitizing the gills of *Hoplerythrinus unitaeniatus* (Spix & Agassiz, 1829), from the Pindaíba River, Cocalinho municipality, Mato Grosso state, Brazil. Scale: 50 μm

A total of ten specimens of *W. caetei* (Fig. 1a, b; Table 1) and seventeen specimens of *Whittingtonocotyle jeju* Neto, Rodrigues & Domingues, 2015 (Fig. 1c, d; Table 1), collected from five specimens of *H. unitaeniatus* (Characiformes: Erythrinidae) were examined morphologically and prepared as permanent slides for detailed characterization.

The type locality of both species is the Caeté River, municipality of Augusto Corrêa, Pará state, Brazil (1°3′58.21″ S, 46°40′3.65″ W), and specimens examined in the present study were obtained from the Pindaíba River (14°31′39.9″ S, 51°41′38.9″ W), municipality of Cocalinho, Mato Grosso state, Brazil. In all cases, parasites were found parasitizing the gills, with a prevalence of 100% (five of five examined hosts) and an intensity of infection ranging from one to four parasites per host. Voucher specimens of *W. caetei* and *W. jeju* were deposited in the Coleção Helmintológica do Instituto de Biociências de Botucatu (CHIBB) under accession numbers 952–954 L and CHIBB 949–951 L, respectively, and molecular voucher specimens were deposited under accession numbers CHIBB 956–957 L and CHIBB 955 L, respectively.

Morphometric data for both species are summarized in Table 1. In *W. caetei*, specimens from the present study exhibited a body length of 1047 (1022–1076) µm, exceeding values reported for the type locality (Caeté River) and being comparable to, or slightly higher than, those recorded from the Guamá River. Haptor width was 203 (179–229) µm. Ventral and dorsal anchor outer lengths were 39 (35–41) µm and 31 (30–35) µm, respectively. Ventral and dorsal bar widths measured 57 (51–59) µm and 47 (47–58) µm, respectively. The male copulatory organ measured 144 (130–153) µm in total length and consistently exhibited approximately 29 coils.

In *W. jeju*, specimens showed a body length of 1474 (1449–1498) µm, exceeding previous records from the Caeté and Guamá rivers. Haptor width was 129 (104–152) µm. Ventral and dorsal anchor outer lengths were 28 (27–28) µm and 29 (26–31) µm, respectively. Ventral and dorsal bar widths were 35 μm and 41 (40–42) µm, respectively. In both the original description and the present study, hook pair 1 was consistently smaller than hook pairs 2–7. The male copulatory organ measured 122 (117–130) µm and consistently exhibited approximately 19 coils. Taken together, morphometric values obtained in the present study were slightly higher than those reported in the original description, while remaining within a comparable range.

The specimens examined herein conform closely to the original description of *W. caetei* provided by Neto, Rodrigues and Domingues [4]. In both datasets, the body is fusiform with poorly developed cephalic lobes (two pairs), cephalic glands absent, and four eyes with the posterior pair larger than the anterior. The pharynx is spherical to subovate. The male copulatory organ is corkscrew-like, composed of approximately 29 coils, and associated with a sheath-like accessory piece. The vaginal complex comprises a heavily sclerotized, coiled canal with distal dilation and a spherical seminal receptacle. The haptor is subhexagonal, bearing anchors with poorly developed roots, a broadly V-shaped ventral bar, and a dorsal bar with a conspicuous elongate anteromedial process. Hooks are similar in morphology, presenting straight shanks, slightly depressed thumbs, an filamentous hook (FH) loop approximately half the shank length, and hook pair 1 smaller than pairs 2–7. The congruence of these diagnostic characters supports the conspecific assignment of the present material to *W. caetei*.

The specimens identified as *W. jeju* in the present study also exhibited a high degree of morphological congruence with the original description provided by Neto, Rodrigues and Domingues [4]. In both datasets, the body is fusiform with inconspicuous cephalic lobes, four eyes with the posterior pair larger and more widely spaced than the anterior pair, and four to five pairs of head organs. Cephalic glands are positioned laterally or postero-laterally to the pharynx, which is ovate and muscular. The male copulatory organ is corkscrew-like, composed of approximately 19 coils, bearing a sclerotized basal cap and associated with a sheath-like accessory piece enclosing the distal rings. The vaginal complex consists of a soft vestibule and a heavily sclerotized sigmoid canal, and the seminal receptacle is pyriform. The peduncle is short and bears haptoral glands that are distally convolute and terminate near the anchor–bar complexes. The haptor is subtrapezoidal to oval in outline. Anchors are similar in both datasets, presenting a broad base, poorly developed roots covered by sclerotized caps, short evenly curved shafts, and distinct points. The ventral bar is curved with tapered ends, whereas the dorsal bar is straight and bears a short anteromedial process approximately one-third of the bar length. Hooks are uniform, with elongate straight shanks, slightly erect thumbs, short points, absence of an FH loop, and hook pair 1 smaller than pairs 2–7. The overall agreement in these diagnostic characters supports the conspecific assignment of the specimens analyzed herein to *W. jeju*.

**Table 1:** Measurements (in µm; mean followed by range and total number of analyzed specimens) of *Whittingtonocotyle caetei* Neto, Rodrigues & Domingues, 2015 and *Whittingtonocotyle Jeju* Neto, Rodrigues & Domingues, 2015 from the present study and related references.

	*Whittingtocotyle caetei*	*Whittingtocotyle caetei*	*Whittingtocotyle caetei*	*Whittingtocotyle jeju*	*Whittingtocotyle jeju*	*Whittingtocotyle jeju*
	Neto, Rodrigues & Domingues, 2015	Neto, Rodrigues & Domingues, 2015	Present study	Neto, Rodrigues & Domingues, 2015	Neto, Rodrigues & Domingues, 2015	Present study
	Caeté River	Guamá River		Caeté River	Guamá River	
*Body*						
Length	605 (550–860) (n = 5)	978 (700–1280) (n = 8)	1047 (1022–1076) (n = 5)	756 (515–1041) (n = 7)	914 (773–1235) (n = 7)	1474 (1498–1449) (n = 5)
Width	241 (146–259) (n = 5)	244 (146–391) (n = 12)	564 (491–607) (n = 5)	181 (138–216) (n = 7)	172 (113–295) (n = 7)	392 (315–468) (n = 5)
*Haptor*						
Length	64 (58–69) (n = 5)	59 (50–65) (n = 8)	114 (111–140) (n = 5)	45 (41–48) (n = 6)	109 (92–126) (n = 6)	83 (80–84) (n = 5)
Width	108 (80–125) (n = 5)	103 (75–134) (n = 10)	203 (179–229) (n = 5)	84 (71–121) (n = 6)	105 (65–152) (n = 6)	129 (104–152) (n = 5)
*Ventral Anchor*						
Outer	34 (33–35) (n = 3)	35 (32–37) (n = 7)	39 (35–41) (n = 4)	26 (25–27) (n = 4)	27 (25–28) (n = 2)	28 (27–28) (n = 4)
Inner	28 (27–30) (n = 3)	27 (26–28) (n = 5)	27 (22–28) (n = 4)	20 (19–21) (n = 3)	21 (20–22) (n = 2)	17 (15–22) (n = 3)
Base	12 (11–12) (n = 3)	14 (13–15) (n = 7)	23 (21–26) (n = 4)	19 (17–20) (n = 2)	–	16 (14–20) (n = 3)
*Dorsal Anchor*						
Outer	31 (29–33) (n = 3)	33 (30–34) (n = 6)	31 (30–35) (n = 3)	27 (25–28) (n = 4)	25 (n = 1)	29 (26–31) (n = 4)
Inner	21 (20–23) (n = 3)	22 (20–24) (n = 6)	21 (20–23) (n = 3)	20 (19–21) (n = 3)	20 (n = 1)	22 (13–23) (n = 3)
Base	16 (16–17) (n = 3)	14 (12–15) (n = 7)	16 (12–18) (n = 4)	19 (17–20) (n = 2)	–	18 (15–20) (n = 4)
*Ventral Bar*						
Length	–	–	6 (4–6) (n = 4)	5 (n = 3)	5 (n = 1)	7 (5–7) (n = 4)
Width	39 (35–42) (n = 3)	43 (42–50) (n = 5)	57 (51–59) (n = 4)	34 (32–36) (n = 4)	31 (n = 1)	35 (n = 3)
*Dorsal Bar*						
**Length	15 (12–17) (n = 3)	14 (12–16) (n = 4)	17 (16–21) (n = 3)	10 (n = 3)	10 (n = 1)	17 (15–18) (n = 4)
Width	39 (35–45) (n = 3)	41 (39–46) (n = 5)	47 (47–58) (n = 4)	33 (29–34) (n = 4)	30 (n = 1)	41 (40–42) (n = 3)
Hook pair 1	9 (n = 1)	8 (n = 1)	9 (9–10) (n = 3)	8 (8–9) (n = 3)	9 (n = 2)	8 (8–10) (n = 3)
Hook pair 2–7	13 (12–14) (n = 10)	12 (11–13) (n = 10)	11 (10–12) (n = 3)	13 (13–14) (n = 7)	13 (12–14) (n = 5)	12 (10–14) (n = 4)
MCO	117 (104–126) (n = 5)	110 (100–120) (n = 10)	144 (130–153) (n = 5)	104 (103–108) (n = 4)	111 (109 –113) (n = 5)	122 (117–130) (n = 5)
Accessory piece	42 (39–45) (n = 3)	–	52 (49–54) (n = 5)	26 (18–28) (n = 4)	23 (22–24) (n = 2)	46 (42–49) (n = 5)

**Dorsal bar length measurement at level of anteromedial process

### Molecular Analyses

**Fig. 2:**
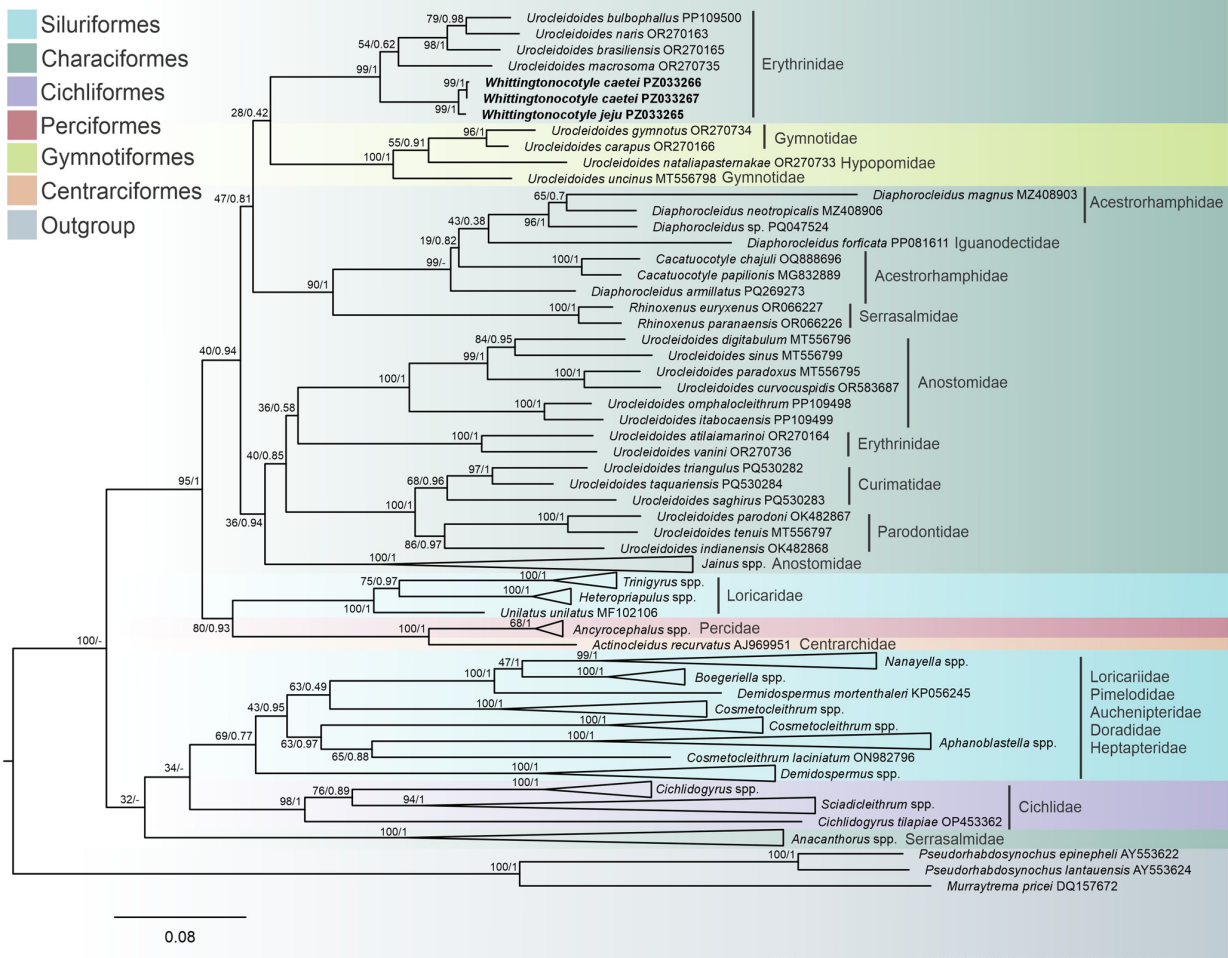
Maximum likelihood topology based on partial sequences of LSU rDNA showing the position of *Whittingtonocotyle caetei* Neto, Rodrigues & Domingues, 2015 and *Whittingtonocotyle jeju* Neto, Rodrigues & Domingues, 2015 (in bold) among other closely related monopisthocotyls. GenBank accession numbers follow species names. The support values are included before or after the nodes as follows: bootstraps for the Maximum Likelihood analyses/posterior probabilities for Bayesian Inference. The branch length scale bar indicates the number of substitutions per site.

Three LSU rDNA sequences were generated in the present study (*W*. *jeju* PZ033265, *W*. *caetei* PZ033266 and PZ033267) and two COI mtDNA sequences (*W*.* jeju *PZ044783 and *W*. *caetei *PZ044784).In both Bayesian inference (BI) and Maximum Likelihood (ML) analyses based on LSU rDNA, *W. caetei* and *W. jeju* consistently clustered together, forming a strongly supported monophyletic unit. Within the taxon sampling adopted in this study, this clade was recovered in close phylogenetic proximity to *Urocleidoides* species parasitizing erythrinid fishes; however, support for deeper backbone relationships was generally low. Pairwise genetic distances supported the close relationship between *W. caetei* and *W. jeju* (1%, corresponding to approximately 8–9 nucleotide differences across the 842 bp LSU alignment), whereas higher divergence was observed relative to erythrinid parasitizing *Urocleidoides *(15–19%). More distant lineages, including *Urocleidoides atilaiamarinoi* Santos Neto & Domingues, 2023 and *Urocleidoides vanini* Santos Neto & Domingues, 2023, showed divergences above 30%, whereas gymnotiform-associated *Urocleidoides* exhibited intermediate values (23–29%). Although *Urocleidoides* terminals were distributed across multiple subclades, *Whittingtonocotyle* was consistently recovered as a cohesive unit across analyses (Fig. 2).

**Fig. 3:**
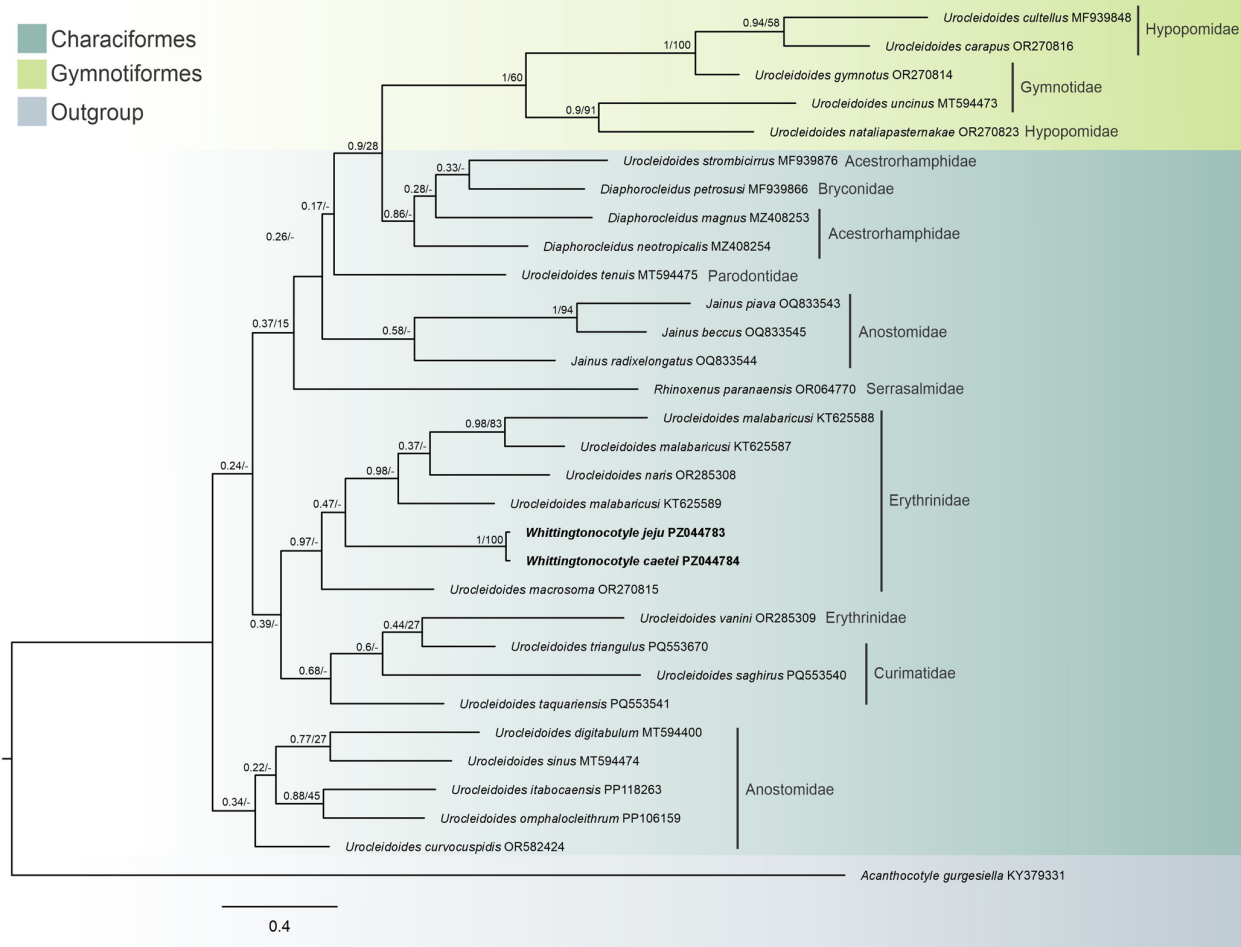
Bayesian inference topology based on partial sequences of COI mtDNA showing the position of *Whittingtonocotyle caetei* Neto, Rodrigues & Domingues, 2015 and *Whittingtonocotyle jeju* Neto, Rodrigues & Domingues, 2015 (in bold) among other closely related monopisthocotyls. GenBank accession numbers follow species names. The support values are included before or after the nodes as follows: posterior probabilities for Bayesian Inference/bootstraps for the Maximum Likelihood analyses. The branch length scale bar indicates the number of substitutions per site.

Similarly, BI and ML analyses based on COI mtDNA recovered *W. caetei* and *W. jeju* as a strongly supported monophyletic unit, again exhibiting low intraspecific divergence (1%, corresponding to approximately 5 nucleotide differences across the 496 bp COI alignment). Overall nodal resolution in the COI topology was limited (Fig. 3), but the congruent recovery of *Whittingtonocotyle* across both molecular markers supports its phylogenetic cohesion within the scope of the present sampling.

## Discussion

The LSU rDNA and COI mtDNA analyses consistently recovered *W. caetei* and *W. jeju* as a strongly supported monophyletic unit across inference methods, providing the first molecular evidence for the phylogenetic cohesion of *Whittingtonocotyle* within Dactylogyridae. Pairwise genetic distances further corroborated this pattern, revealing low divergence between *W. caetei* and *W. jeju* (1%, corresponding to approximately 8–9 nucleotide differences across the 842 bp LSU alignment and ~ 5 nucleotide differences across the 496 bp COI alignment), which is consistent with their close evolutionary relationship and shared generic identity.

However, beyond the monophyly of *Whittingtonocotyle*, relationships among major dactylogyrid lineages were weakly resolved, particularly in the LSU rDNA topology, where several internal nodes exhibited low support. This limited resolution is consistent with the conservative evolutionary rate of LSU rDNA for resolving recent divergences, uneven taxon sampling imposed by the availability of homologous sequences in public databases, and the inclusion of distantly related taxa, which may increase alignment complexity and reduce nodal support. Consequently, deeper intergeneric relationships inferred here should be interpreted cautiously and were not the primary focus of this study.

In this context, the LSU rDNA reconstruction recovered *Whittingtonocotyle* in phylogenetic proximity to terminals identified as *Urocleidoides* parasitizing erythrinid hosts, however, support for the relevant backbone nodes was weak. Genetic divergence values indicate a substantially deeper separation between *Whittingtonocotyle* and erythrinid-associated *Urocleidoides* (15–19%), and even higher divergence relative to *U. atilaiamarinoi* and *U. vanini* (> 30%), whereas gymnotiform-associated *Urocleidoides* exhibited intermediate divergence levels (23–29%). Taken together, these results suggest that the observed proximity reflects a preliminary phylogenetic signal rather than a robust sister-group relationship. Moreover, dactylogyrids parasitizing Erythrinidae did not form a monophyletic assemblage in the LSU topology, indicating that host-family association alone is insufficient to predict evolutionary relationships.

Similarly, the COI mtDNA phylogeny exhibited overall low resolution, with numerous unsupported internal nodes. This outcome is expected when mitochondrial protein-coding markers are applied beyond shallow phylogenetic levels, as substitution saturation and homoplasy may reduce phylogenetic signal at deeper nodes. Additionally, COI sampling for Dactylogyridae remains sparse and taxonomically uneven. In this context, COI is more informative for assessing genetic cohesion and divergence among closely related taxa than for resolving deep phylogenetic structure. Accordingly, in the present study, the COI dataset primarily corroborates the low genetic divergence between *W. caetei* and *W. jeju* and supports their cohesion as a genus-level unit.

Complementing the molecular evidence, morphological and morphometric data provide an independent and concordant line of support for the taxonomic stability of *Whittingtonocotyle*, as the monophyletic clustering recovered in the molecular analyses is mirrored by the high morphological similarity observed between the two species.

Although populations appeared to differ slightly in overall body size, specimens examined in the present study remained highly similar to those previously reported from the Caeté and Guamá rivers [4] (Table 1), particularly in *W. jeju*, which showed larger values for total length and dimensions of sclerotized structures. These size differences were accompanied by proportional scaling of haptoral and copulatory structures, indicating population-level and size-related variation rather than discrete morphological divergence. In addition, minor discrepancies in absolute measurements may also reflect methodological factors, such as specimen flattening and mounting procedures, which are known to influence morphometric values in monopisthocotyls.

Despite relatively small differences in absolute measurements among populations, the dimensions and configuration of some important structures (including anchors, bars and hooks) showed broad overlap across datasets. In both species, the number of coils of the male copulatory organ, the general morphology of the copulatory complex, bars, anchors and hook morphology remained consistent with the original descriptions. These patterns indicate that the morphometric variation observed among specimens from the Caeté River, Guamá River, and Pindaíba River likely reflects geographic and ontogenetic variation rather than taxonomic differentiation.

Finally, although the discussion necessarily builds upon the phylogenetic framework generated in the present study, broader evolutionary inferences within Dactylogyridae remain constrained by the limited resolution of available datasets and by the scarcity of comparable multilocus data for Neotropical lineages. At present, few studies integrate morphology and molecular data for erythrinid-associated dactylogyrids, which restricts direct comparative analyses. Therefore, references to previous works, including Neto and Domingues [27], are used primarily as contextual benchmarks rather than as exclusive interpretative foundations.

Overall, this study provides the first molecular data for *Whittingtonocotyle* and demonstrates the consistent monophyly of the genus across independent markers and analytical approaches. While the present dataset is not intended to resolve higher-level phylogenetic relationships within Dactylogyridae, it establishes a robust molecular framework for *Whittingtonocotyle* and highlights the need for broader taxon sampling and multilocus datasets to improve phylogenetic resolution among Neotropical freshwater dactylogyrids.

## Supplementary Information

Below is the link to the electronic supplementary material.

Supplementary Material 1

Supplementary Material 2

Supplementary Material 3

## Data Availability

All data will be available for consultation. Whenever necessary, they should be requested from the corresponding author. The specimens used for this work will be available in specialized scientific collections.
