# Supplementary material for: Phylogenetic Placement of Whittingtonocotyle Neto, Rodrigues & Domingues, 2015 (Monopisthocotyla: Dactylogyridae) Inferred from the First Molecular Data of Both Described Species
Source: Acta Parasitol. 2026 Mar 9;71(2):59. doi: 10.1007/s11686-026-01239-8 (PMC12971823; doi:10.1007/s11686-026-01239-8)
Supplement: Supplementary file 1 — Supplementary Material 1 [file 11686_2026_1239_MOESM1_ESM.docx]

| **Monogenea species** | **Host species** | **Locality** | **Genbank ID** | | **References** |  |
| --- | --- | --- | --- | --- | --- | --- |
|  |  |  | **28S rDNA** | **COI mtDNA** |  |  |
| *Acanthocotyle gurgesiella* Ñacari, Sepulveda, Escribano & Oliva, 2017 | *Gurgesiella furvescens* de Buen, 1959 | Chile | - | KY379331 | Ñacari *et al*. 2018 | |
| *Actinocleidus recurvatus* Mizelle & Donahue, 1944 | *Lepomis gibbosus* (Linnaeus, 1758) | Slovak Republic | AJ969951 | - | Šimková *et al*. 2006 | |
| *Anacanthorus lepyrophallus* Kritsky, Boeger & Van Every, 1992 | *Serrasalmus maculatus*Kner, 1858 | Brazil | MH843718 | - | Moreira *et al*. 2019 | |
| *Anacanthorus maltai* Boeger & Kritsky, 1988 | *Pygocentrus nattereri* Kner, 1858 | Brazil | MH843716 | - | Moreira *et al*. 2019 | |
| *Anacanthorus penilabiatus* Boeger, Husak & Martins, 1995 | *Piaractus mesopotamicus*(Holmberg, 1887) | Brazil | MH843719 | - | Moreira *et al*. 2019 | |
| *Anacanthorus rondonensis* Boeger & Kritsky, 1988 | *Pygocentrus nattereri*Kner, 1858 | Brazil | MH843714 | - | Moreira *et al*. 2019 | |
| *Ancyrocephalus paradoxus* Creplin, 1839 | *Sander lucioperca* (Linnaeus, 1758) | Czech Republic | AJ969952 | - | Šimková *et al*. 2006 | |
| *Ancyrocephalus percae* Ergens, 1966 | *Perca fluviatilis* Linnaeus, 1758 | Germany | KF499080 | - | Behrmann-Godel *et al*. 2014 | |
| *Aphanoblastella aurorae* Mendoza-Palmero, Scholz, Mendoza-Franco & Kuchta, 2012 | *Goeldiella eques* (Müller & Troschel, 1849) | Peru | KP056239 | - | Mendoza-Palmero *et al*. 2015 | |
| *Aphanoblastella magna* Yamada, Acosta, Yamada, Scholz & Silva, 2018 | *Pimelodella avanhandavae* Eigenmann, 1917 | Brazil | MH688484 | - | Yamada *et al*. 2018 | |
| *Aphanoblastella travassosi*(Price, 1938) | *Rhamdia guatemalensis* (Günther, 1864) | Mexico | MK358458 | - | Mendoza-Palmero *et al*. 2015 | |
| *Boegeriella conica* (Mendoza-Palmero, Mendoza-Franco, Acosta & Scholz, 2019) | *Platynematichthys notatus* (Jardine, 1841) | Peru | MK834513 | - | Mendoza-Palmero *et al*. 2019 | |
| *Boegeriella ophiocirrus* (Mendoza-Palmero, Mendoza-Franco, Acosta & Scholz, 2019) | *Platystomatichthys sturio* (Kner, 1858) | Peru | MK834515 | - | Mendoza-Palmero *et al*. 2019 | |
| *Cacatuocotyle chajuli* Mendoza-Franco, Caspeta-Mandujano & Salgado-Maldonado, 2013 | *Astyanax mexicanus* (De Filippi, 1853) | Mexico | OQ888696 | - | Santa-Cruz *et al*. 2023 | |
| *Cacatuocotyle papilionis* Zago, Franceschini, Müller & Silva, 2018 | *Astyanax lacustris* (Lütken, 1875) | Brazil | MG832889 | - | Zago *et al*. 2018 | |
| *Cichlidogyrus halli* (Price & Kirk, 1967) | - | Brazil | KX869729 | - | Unpublished | |
| *Cichlidogyrus mbirizei* Muterezi Bukinga, Vanhove, Van Steenberge & Pariselle, 2012 | - | Brazil | MG030378 | - | Unpublished | |
| *Cichlidogyrus tilapiae*  Paperna, 1960 | *Oreochromis mossambicus* (Peters, 1852) | China | OP453362 | - | Unpublished | |
| *Cosmetocleithrum bifurcum* Mendoza-Franco, Mendoza-Palmero & Scholz, 2016 | *Hassar orestis* (Steindachner, 1875) | Peru | KP056217 | - | Mendoza-Palmero *et al*. 2015 | |
| *Cosmetocleithrum bulbocirrus* Kritsky, Thatcher& Boeger, 1986 | *Pterodoras granulosus* (Valenciennes, 1821) | Brazil | MG001326 | - | Acosta *et al*. 2018 | |
| *Cosmetocleithrum gigas* Morey, Cachique & Babilonia, 2020 | *Oxydoras niger* (Valenciennes, 1821) | Peru | ON982794 | - | Mendoza-Palmero *et al*. 2022 | |
| *Cosmetocleithrum gussevi* Kritsky, Thatcher & Boeger, 1986 | *Oxydoras niger* (Valenciennes, 1821) | Peru | ON982795 | - | Mendoza-Palmero *et al*. 2022 | |
| *Cosmetocleithrum laciniatum* Yamada, Yamada, Silva & Anjos, 2017 | *Trachelyopterus galeatus* (Linnaeus, 1766) | Peru | ON982796 | - | Mendoza-Palmero *et al*. 2022 | |
| *Cosmetocleithrum rarum* Kritsky, Thatcher & Boeger, 1986 | *Oxydoras niger* (Valenciennes, 1821) | Peru | ON982797 | - | Mendoza-Palmero *et al*. 2022 | |
| *Demidospermus anus* Suriano, 1983 | *Loricariichthys platymetopon*Isbrücker & Nijssen, 1979 | Brazil | KY766957 | - | Franceschini *et al*. 2017 | |
| *Demidospermus prolixus* Franceschini, Zago, Müller, Francisco, Takemoto & Silva, 2017 | *Proloricaria prolixa* (Isbrücker & Nijssen, 1978) | Brazil | KY766955 | - | Franceschini *et al*. 2017 | |
| *Demidospermus rhinelepisi* Acosta, Scholz, Blasco-Costa, Alves & Silva, 2018 | *Rhinelepis aspera* Spix & Agassiz, 1829 | Brazil | MG001324 | - | Acosta *et al*. 2018 | |
| *Demidospermus spirophallus* Franceschini, Zago, Müller, Francisco, Takemoto & Silva, 2017 | *Proloricaria prolixa* (Isbrücker & Nijssen, 1978) | Brazil | KY766954 | - | Franceschini *et al*. 2017 | |
| *Diaphorocleidus* sp. | *Psalidodon fasciatus (Cuvier, 1819)* | Brazil | PQ047524 | - | Unpublished | |
| *Diaphorocleidus armillatus*  Jogunoori, Kritsky & Venkatanarasaiah, 2004 | *Gymnocorymbus ternetzi*  (Boulenger, 1895) | India | PQ269273 | - | Tripathi *et al*. 2025 | |
| *Diaphorocleidus forficata* Santos-Neto, Paixão & Domingues, 2024 | *Bryconops melanurus* (Bloch, 1794) | Brazil | PP081611 | - | Santos-Neto, Paixão and Domingues, 2024 | |
| *Diaphorocleidus magnus* Zago, Franceschini, Abdallah, Müller, Azevedo & Silva, 2021 | *Astyanax lacustris* (Lütken, 1875) | Brazil | MZ408903 | MZ408253 | Zago *et al*. 2021 | |
| *Diaphorocleidus neotropicalis* Zago, Franceschini, Abdallah, Müller, Azevedo & Silva, 2021 | *Astyanax lacustris* (Lütken, 1875) | Brazil | MZ408906 | MZ408254 | Zago *et al*. 2021 | |
| *Diaphorocleidus petrosusi* Mendoza-Franco, Aguirre-Macedo & Vidal-Martínez, 2007 | *Brycon petrosus*Meek and Hildebrand, 1913 | Panama | - | MF939866 | Unpublished | |
| *Heteropriapulus anchoradiatus* Acosta, Franceschini, Zago, Scholz & Silva, 2017 | *Pterygoplichthys ambrosettii* (Holmberg, 1893) | Brazil | MF116371 | - | Acosta *et al*. 2017 | |
| *Heteropriapulus heterotylus* Jogunoori, Kritsky & Venkatanarasaiah, 2004 | *Pterygoplichthys ambrosettii* (Holmberg, 1893) | Brazil | MF116370 | - | Acosta *et al*. 2017 | |
| *Heteropriapulus simplex* Li & Huang, 2012 | *Pterygoplichthys ambrosettii* (Holmberg, 1893) | Brazil | MF116372 | - | Acosta *et al*. 2017 | |
| *Jainus beccus* Yamada, Müller, Zago, Yamada, Ebert, Franceschini & Silva, 2023 | *Leporinus friderici* (Bloch, 1794) | Brazil | - | OQ833545 | Yamada *et al*. 2023 | |
| *Jainus piava* Karling, Bellay, Takemoto & Pavanelli, 2011 | *Schizodon nasutus* Kner, 1858 | Brazil | OQ843019 | OQ833543 | Yamada *et al*. 2023 | |
| *Jainus radixelongatus*  Yamada, Müller, Zago, Yamada, Ebert, Franceschini & Silva, 2023 | *Leporinus striatus* Kner, 1858 | Brazil | OQ843018 | OQ833544 | Yamada *et al*. 2023 | |
| *Murraytrema pricei* Bychowsky & Nagibina, 1977 | *Nibea albiflora* Richardson, 1846 | China | DQ157672 | - | Wu *et al*. 2006 | |
| *Nanayella amplofalcis* Acosta, Mendoza-Palmero, Silva & Scholz, 2019 | *Sorubim lima* (Bloch & Schneider, 1801) | Brazil | MG001325 | - | Acosta *et al*. 2019 | |
| *Nanayella fluctuatrium* Acosta, Mendoza-Palmero, Silva & Scholz, 2019 | *Hemisorubim platyrhynchos* (Valenciennes, 1840) | Brazil | MG001327 | - | Acosta *et al*. 2019 | |
| *Nanayella processusclavis* Acosta, Mendoza-Palmero, Silva & Scholz, 2019 | *Hemisorubim platyrhynchos* (Valenciennes, 1840) | Brazil | MG001328 | - | Acosta *et al*. 2019 | |
| *Pseudorhabdosynochus epinepheli* (Yamaguti, 1938) | *Epinephelus bruneus* Bloch, 1793 | China | AY553622 | - | Wu *et al*. 2005 | |
| *Pseudorhabdosynochus lantauensis* (Beverley-Burton & Suriano, 1981) | *Epinephelus bruneus* Bloch, 1793 | China | AY553624 | - | Wu *et al*. 2005 | |
| *Rhinoxenus euryxenus* Dominngues & Boeger, 2005 | *Serrasalmus marginatus* Valenciennes, 1837 | Brazil | OR066227 | - | Osaki-Pereira *et al*. 2023 | |
| *Rhinoxenus paranaensis* Rossin & Timi, 2019 | *Serrasalmus maculatus*Kner, 1858 | Brazil | OR066226 | OR064770 | Osaki-Pereira *et al*. 2023 | |
| *Sciadicleithrum meekii* Mendoza-Franco, Scholz & Vidal-Martínez, 1997 | *Thorichthys meeki*(Brind, 1918) | Mexico | KY305889 | - | Mendoza-Palmero *et al*. 2017 | |
| *Sciadicleithrum mexicanum* Kritsky, Vidal-Martínez & Rodríguez-Canul, 1994 | *Rocio octofasciata* (Regan, 1903) | Mexico | KY305886 | - | Mendoza-Palmero *et al*. 2017 | |
| *Sciadicleithrum splendidae* Kritsky, Vidal-Martínez & Rodríguez-Canul, 1994 | *Parachromis friedrichsthalii*(Heckel, 1840) | Mexico | KY305890 | - | Mendoza-Palmero *et al*. 2017 | |
| *Trinigyrus anthus* Franceschini, Acosta, Zago, Müller & Silva, 2020 | *Hypostomus regani* (Ihering, 1905) | Brazil | MN947622 | - | Franceschini *et al*. 2020 | |
| *Trinigyrus carvalhoi* Franceschini, Acosta, Zago, Müller & Silva, 2020 | *Hypostomus ancistroides* (Ihering, 1911) | Brazil | MN947608 | - | Franceschini *et al*. 2020 | |
| *Trinigyrus peregrinus* Nitta & Nagasawa, 2016 | *Pterygoplichthys ambrosettii* (Holmberg, 1893) | Brazil | MN944890 | - | Franceschini *et al*. 2020 | |
| *Unilatus unilatus* Mizelle & Kritsky, 1967 | *Pterygoplichthys ambrosettii* (Holmberg, 1893) | Brazil | MF102106 | - | Acosta *et al*. 2018 | |
| *Urocleidoides atialaiamarinoi* Santos Neto & Domingues, 2023 | *Hoplerytrinus unitaeniatus* (Spix & Agassiz, 1829) | Brazil | OR270164 | - | Santos Neto and Domingues 2023 | |
| *Urocleidoides brasiliensis* Rosim, Mendoza-Franco & Luque, 2011 | *Hoplias malabaricus* (Bloch, 1794) | Brazil | OR270165 | - | Santos Neto and Domingues 2023 | |
| *Urocleidoides carapus* Mizelle, Kritsky & Crane, 1968 | *Gymnotus carapo* Linnaeus, 1758 | Brazil | OR270166 | OR270816 | Santos Neto and Domingues 2023 | |
| *Urocleidoides cultellus* Mendoza-Franco & Reina, 2008 | - | Panama | - | MF939848 | Unpublished | |
| *Urocleidoides curvocuspidis* Ebert, Osaki-Pereira & Silva, 2023 | *Schizodon nasutus* Kner, 1858 | Brazil | OR583687 | OR582424 | Ebert *et al*. 2023 | |
| *Urocleidoides digitabulum* Zago, Yamada, Yamada, Franceschini, Bongiovani & Silva, 2020 | *Megaleporinus elongatus* (Valenciennes, 1850) | Brazil | MT556796 | MT594400 | Zago *et al*. 2020 | |
| *Urocleidoides gymnotus*  Mizelle, Kritsky & Crane, 1968 | *Gymnotus carapo* Linnaeus, 1758 | Brazil | OR270734 | OR270814 | Santos Neto and Domingues 2023 | |
| *Urocleidoides indianensis* Oliveira, Silva, Vieira & Acosta, 2021 | *Parodon nasus* Kner, 1859 | Brazil | OK482868 | - | Oliveira *et al*. 2021 | |
| *Urocleidoides itabocaensis* Santos-Neto & Domingues, 2024 | *Pseudanos trimaculatus* (Kner, 1858) | Brazil | PP109499 | PP118263 | Santos-Neto and Domingues, 2024 | |
| *Urocleidoides macrosoma* Santos Neto & Domingues, 2023 | *Hoplias malabaricus* (Bloch, 1794) | Brazil | OR270735 | OR270815 | Santos Neto and Domingues 2023 | |
| *Urocleidoides malabaricusi*  Rosim, Mendoza-Franco & Luque, 2011 | *Hoplias* aff. *malabaricus* (Bloch, 1794) | Brazil | - | KT625587  KT625588  KT625589 | Gasques *et al*. 2016 | |
| *Urocleidoides naris*  Rosim, Mendoza-Franco & Luque, 2011 | *Hoplias malabaricus* (Bloch, 1794) | Brazil | OR270163 | OR285308 | Santos Neto and Domingues 2023 | |
| *Urocleioides nataliapasternakae* Santos Neto & Domingues, 2023 | *Brachyhypopomus brevirostris* | Brazil | OR270733 | OR270823 | Santos Neto and Domingues 2023 | |
| *Urocleidoides omphalocleithrum* Santos-Neto & Domingues, 2024 | *Pseudanos trimaculatus* (Kner, 1858) | Brazil | PP109498 | PP106159 | Santos-Neto and Domingues, 2024 | |
| *Urocleidoides parodoni* Oliveira, Silva, Vieira & Acosta, 2021 | *Parodon nasus* Kner, 1859 | Brazil | OK482867 | - | Oliveira *et al*. 2021 | |
| *Urocleidoides paradoxus* Kritsky, Thatcher & Boeger, 1986 | *Leporinus friderici* (Bloch, 1794) | Brazil | MT556795 | - | Zago *et al*. 2020 | |
| *Urocleidoides saghirus* Yamada, Osaki-Pereira & Silva, 2024 | *Cyphocharax modestus* (Fernández-Yépez, 1948) | Brazil | PQ530283 | PQ553540 | Yamada *et al*. 2024 | |
| *Urocleidoides sinus* Zago, Yamada, Yamada, Franceschini, Bongiovani & Silva, 2020 | *Schizodon nasutus* Kner, 1858 | Brazil | MT556799 | MT594474 | Zago *et al*. 2020 | |
| *Urocleidoides strombicirrus* (Price and Bussing, 1967) | - | Panama | - | MF939876 | Unpublished | |
| *Urocleidoides taquariensis* Yamada, Osaki-Pereira & Silva, 2024 | *Cyphocharax modestus* (Fernández-Yépez, 1948) | Brazil | PQ530284 | PQ553541 | Yamada *et al*. 2024 | |
| *Urocleidoides tenuis* Zago, Yamada, Yamada, Franceschini, Bongiovani & Silva, 2020 | *Apareiodon piracicabae* (Eigenmann, 1907) | Brazil | MT556797 | MT594475 | Zago *et al*. 2020 | |
| *Urocleidoides triangulus* Rossin & Timi, 2016 | *Cyphocharax modestus* (Fernández-Yépez, 1948) | Brazil | PQ530282 | PQ553670 | Yamada *et al*. 2024 | |
| *Urocleidoides uncinus* Zago, Yamada, Yamada, Franceschini, Bongiovani & Silva, 2020 | *Gymnotus inaequilabiatus* (Valenciennes, 1839*)* | Brazil | MT556798 | MT594473 | Zago *et al*. 2020 | |
| *Urocleidoides vanini* Santos Neto & Domingues, 2023 | *Erythrinus erythrinus* (Bloch & Schneider, 1801) | Brazil | OR270736 | OR285309 | Santos Neto and Domingues 2023 | |
| ***Whittingtonocotyle caetei* Neto, Rodrigues & Domingues, 2015** | ***Hoplerytrinus unitaeniatus* (Spix & Agassiz, 1829)** | **Brazil** | **PZ033266**  **PZ033267** | **PZ044784** | **Present study** | |
| ***Whittingtonocotyle jeju* Neto, Rodrigues & Domingues, 2015** | ***Hoplerytrinus unitaeniatus* (Spix & Agassiz, 1829)** | **Brazil** | **PZ033265** | **PZ044783** | **Present study** | |
