# Supplementary material for: Phylogenetic Placement of Whittingtonocotyle Neto, Rodrigues & Domingues, 2015 (Monopisthocotyla: Dactylogyridae) Inferred from the First Molecular Data of Both Described Species
Source: Acta Parasitol. 2026 Mar 9;71(2):59. doi: 10.1007/s11686-026-01239-8 (PMC12971823; doi:10.1007/s11686-026-01239-8)
Supplement: Supplementary file 2 — Supplementary Material 2 [file 11686_2026_1239_MOESM2_ESM.docx]

|  |  | **1** | **2** | **3** | 4 | 5 | 6 | 7 | 8 | 9 | 10 | 11 | 12 | 13 | 14 | 15 | 16 | 17 | 18 | 19 | 20 | 21 | 22 | 23 | 24 | 25 | 26 | 27 | 28 | 29 | 30 | 31 | 32 | 33 | 34 | 35 | 36 | 37 | 38 | 39 |
| --- | --- | --- | --- | --- | --- | --- | --- | --- | --- | --- | --- | --- | --- | --- | --- | --- | --- | --- | --- | --- | --- | --- | --- | --- | --- | --- | --- | --- | --- | --- | --- | --- | --- | --- | --- | --- | --- | --- | --- | --- |
| **1** | ***Whittingtonocotyle jeju* PZ033265** |  |  |  |  |  |  |  |  |  |  |  |  |  |  |  |  |  |  |  |  |  |  |  |  |  |  |  |  |  |  |  |  |  |  |  |  |  |  |  |
| **2** | ***Whittingtonocotyle caetei* PZ033266** | **0,01** |  |  |  |  |  |  |  |  |  |  |  |  |  |  |  |  |  |  |  |  |  |  |  |  |  |  |  |  |  |  |  |  |  |  |  |  |  |  |
| **3** | ***Whittingtonocotyle caetei* PZ033267** | **0,01** | **0,00** |  |  |  |  |  |  |  |  |  |  |  |  |  |  |  |  |  |  |  |  |  |  |  |  |  |  |  |  |  |  |  |  |  |  |  |  |  |
| 4 | *Urocleidoides vanini* OR270736 | **0,32** | **0,32** | **0,32** |  |  |  |  |  |  |  |  |  |  |  |  |  |  |  |  |  |  |  |  |  |  |  |  |  |  |  |  |  |  |  |  |  |  |  |  |
| 5 | *Urocleidoides atilaiamarinoi* OR270164 | **0,31** | **0,31** | **0,31** | 0,12 |  |  |  |  |  |  |  |  |  |  |  |  |  |  |  |  |  |  |  |  |  |  |  |  |  |  |  |  |  |  |  |  |  |  |  |
| 6 | *Urocleidoides macrosoma* OR270735 | **0,17** | **0,17** | **0,17** | 0,22 | 0,21 |  |  |  |  |  |  |  |  |  |  |  |  |  |  |  |  |  |  |  |  |  |  |  |  |  |  |  |  |  |  |  |  |  |  |
| 7 | *Urocleidoides brasiliensis* OR270165 | **0,16** | **0,16** | **0,16** | 0,21 | 0,21 | 0,09 |  |  |  |  |  |  |  |  |  |  |  |  |  |  |  |  |  |  |  |  |  |  |  |  |  |  |  |  |  |  |  |  |  |
| 8 | *Urocleidoides naris* OR270163 | **0,19** | **0,18** | **0,18** | 0,21 | 0,22 | 0,11 | 0,06 |  |  |  |  |  |  |  |  |  |  |  |  |  |  |  |  |  |  |  |  |  |  |  |  |  |  |  |  |  |  |  |  |
| 9 | *Urocleidoides bulbophallus* PP109500 | **0,16** | **0,15** | **0,15** | 0,21 | 0,22 | 0,10 | 0,06 | 0,06 |  |  |  |  |  |  |  |  |  |  |  |  |  |  |  |  |  |  |  |  |  |  |  |  |  |  |  |  |  |  |  |
| 10 | *Cacatuocotyle chajuli* OQ888696 | **0,22** | **0,22** | **0,23** | 0,25 | 0,24 | 0,22 | 0,23 | 0,22 | 0,23 |  |  |  |  |  |  |  |  |  |  |  |  |  |  |  |  |  |  |  |  |  |  |  |  |  |  |  |  |  |  |
| 11 | *Cacatuocotyle papilionis* MG832889 | **0,21** | **0,22** | **0,22** | 0,24 | 0,23 | 0,22 | 0,23 | 0,22 | 0,23 | 0,05 |  |  |  |  |  |  |  |  |  |  |  |  |  |  |  |  |  |  |  |  |  |  |  |  |  |  |  |  |  |
| 12 | *Diaphorocleidus armillatus* PQ269273 | **0,31** | **0,32** | **0,31** | 0,23 | 0,22 | 0,19 | 0,21 | 0,21 | 0,21 | 0,14 | 0,14 |  |  |  |  |  |  |  |  |  |  |  |  |  |  |  |  |  |  |  |  |  |  |  |  |  |  |  |  |
| 13 | *Diaphorocleidus* sp. PQ047524 | **0,22** | **0,22** | **0,22** | 0,34 | 0,34 | 0,30 | 0,32 | 0,33 | 0,33 | 0,16 | 0,16 | 0,21 |  |  |  |  |  |  |  |  |  |  |  |  |  |  |  |  |  |  |  |  |  |  |  |  |  |  |  |
| 14 | *Diaphorocleidus forficata* PP081611 | **0,35** | **0,37** | **0,35** | 0,24 | 0,23 | 0,22 | 0,22 | 0,21 | 0,22 | 0,18 | 0,16 | 0,15 | 0,26 |  |  |  |  |  |  |  |  |  |  |  |  |  |  |  |  |  |  |  |  |  |  |  |  |  |  |
| 15 | *Diaphorocleidus neotropicalis* MZ408906 | **0,21** | **0,21** | **0,22** | 0,22 | 0,22 | 0,19 | 0,21 | 0,22 | 0,22 | 0,13 | 0,13 | 0,14 | 0,09 | 0,17 |  |  |  |  |  |  |  |  |  |  |  |  |  |  |  |  |  |  |  |  |  |  |  |  |  |
| 16 | *Diaphorocleidus magnus* MZ408903 | **0,30** | **0,30** | **0,30** | 0,22 | 0,23 | 0,21 | 0,22 | 0,23 | 0,22 | 0,19 | 0,19 | 0,14 | 0,19 | 0,18 | 0,15 |  |  |  |  |  |  |  |  |  |  |  |  |  |  |  |  |  |  |  |  |  |  |  |  |
| 17 | *Jainus radixelongatus* OQ843018 | **0,24** | **0,23** | **0,24** | 0,25 | 0,24 | 0,21 | 0,22 | 0,23 | 0,23 | 0,20 | 0,21 | 0,26 | 0,27 | 0,26 | 0,19 | 0,26 |  |  |  |  |  |  |  |  |  |  |  |  |  |  |  |  |  |  |  |  |  |  |  |
| 18 | *Jainus piava* OQ843019 | **0,22** | **0,22** | **0,23** | 0,23 | 0,23 | 0,21 | 0,21 | 0,21 | 0,21 | 0,18 | 0,18 | 0,24 | 0,23 | 0,24 | 0,17 | 0,25 | 0,16 |  |  |  |  |  |  |  |  |  |  |  |  |  |  |  |  |  |  |  |  |  |  |
| 19 | *Urocleidoides omphalocleithrum* PP109498 | **0,28** | **0,27** | **0,26** | 0,23 | 0,22 | 0,21 | 0,20 | 0,23 | 0,21 | 0,25 | 0,25 | 0,22 | 0,36 | 0,23 | 0,24 | 0,23 | 0,26 | 0,25 |  |  |  |  |  |  |  |  |  |  |  |  |  |  |  |  |  |  |  |  |  |
| 20 | *Urocleidoides itabocaensis* PP109499 | **0,30** | **0,29** | **0,28** | 0,23 | 0,23 | 0,22 | 0,21 | 0,24 | 0,22 | 0,26 | 0,25 | 0,24 | 0,37 | 0,25 | 0,24 | 0,23 | 0,26 | 0,24 | 0,06 |  |  |  |  |  |  |  |  |  |  |  |  |  |  |  |  |  |  |  |  |
| 21 | *Urocleidoides curvocuspidis* OR583687 | **0,23** | **0,23** | **0,23** | 0,37 | 0,35 | 0,32 | 0,29 | 0,32 | 0,32 | 0,26 | 0,24 | 0,36 | 0,25 | 0,37 | 0,24 | 0,29 | 0,29 | 0,25 | 0,28 | 0,26 |  |  |  |  |  |  |  |  |  |  |  |  |  |  |  |  |  |  |  |
| 22 | *Urocleidoides gymnotu*s OR270734 | **0,26** | **0,25** | **0,26** | 0,22 | 0,21 | 0,17 | 0,18 | 0,18 | 0,19 | 0,21 | 0,21 | 0,19 | 0,29 | 0,21 | 0,19 | 0,20 | 0,22 | 0,20 | 0,20 | 0,20 | 0,32 |  |  |  |  |  |  |  |  |  |  |  |  |  |  |  |  |  |  |
| 23 | *Urocleidoides nataliapasternakae* OR270733 | **0,28** | **0,29** | **0,28** | 0,23 | 0,20 | 0,18 | 0,17 | 0,19 | 0,18 | 0,22 | 0,22 | 0,19 | 0,32 | 0,20 | 0,20 | 0,21 | 0,24 | 0,24 | 0,21 | 0,23 | 0,33 | 0,13 |  |  |  |  |  |  |  |  |  |  |  |  |  |  |  |  |  |
| 24 | *Urocleidoides carapus* OR270166 | **0,27** | **0,27** | **0,26** | 0,21 | 0,20 | 0,16 | 0,17 | 0,19 | 0,18 | 0,21 | 0,21 | 0,19 | 0,31 | 0,20 | 0,19 | 0,20 | 0,22 | 0,21 | 0,21 | 0,21 | 0,32 | 0,04 | 0,10 |  |  |  |  |  |  |  |  |  |  |  |  |  |  |  |  |
| 25 | *Rhinoxenus euryxenus* OR066227 | **0,26** | **0,26** | **0,26** | 0,26 | 0,25 | 0,22 | 0,21 | 0,22 | 0,21 | 0,22 | 0,21 | 0,20 | 0,28 | 0,22 | 0,20 | 0,21 | 0,26 | 0,22 | 0,24 | 0,25 | 0,29 | 0,21 | 0,23 | 0,22 |  |  |  |  |  |  |  |  |  |  |  |  |  |  |  |
| 26 | *Rhinoxenus paranaensis* OR066226 | **0,22** | **0,22** | **0,23** | 0,26 | 0,24 | 0,21 | 0,20 | 0,22 | 0,21 | 0,18 | 0,17 | 0,19 | 0,24 | 0,21 | 0,17 | 0,24 | 0,23 | 0,19 | 0,24 | 0,25 | 0,26 | 0,22 | 0,22 | 0,22 | 0,04 |  |  |  |  |  |  |  |  |  |  |  |  |  |  |
| 27 | *Urocleidoides uncinus* MT556798 | **0,17** | **0,17** | **0,18** | 0,22 | 0,22 | 0,16 | 0,18 | 0,20 | 0,20 | 0,18 | 0,17 | 0,22 | 0,22 | 0,22 | 0,17 | 0,24 | 0,19 | 0,17 | 0,24 | 0,24 | 0,24 | 0,11 | 0,14 | 0,12 | 0,21 | 0,19 |  |  |  |  |  |  |  |  |  |  |  |  |  |
| 28 | *Urocleidoides indianensis* OK482868 | **0,18** | **0,18** | **0,19** | 0,25 | 0,24 | 0,22 | 0,20 | 0,22 | 0,22 | 0,19 | 0,19 | 0,21 | 0,24 | 0,25 | 0,17 | 0,23 | 0,20 | 0,18 | 0,22 | 0,24 | 0,26 | 0,21 | 0,21 | 0,22 | 0,24 | 0,20 | 0,17 |  |  |  |  |  |  |  |  |  |  |  |  |
| 29 | *Urocleidoides parodon*i OK482867 | **0,19** | **0,19** | **0,20** | 0,24 | 0,23 | 0,22 | 0,20 | 0,20 | 0,21 | 0,21 | 0,20 | 0,23 | 0,24 | 0,26 | 0,18 | 0,25 | 0,19 | 0,17 | 0,23 | 0,24 | 0,25 | 0,23 | 0,21 | 0,22 | 0,24 | 0,20 | 0,17 | 0,11 |  |  |  |  |  |  |  |  |  |  |  |
| 30 | *Urocleidoides digitabulum* MT556796 | **0,19** | **0,19** | **0,20** | 0,22 | 0,21 | 0,23 | 0,22 | 0,23 | 0,22 | 0,18 | 0,17 | 0,24 | 0,23 | 0,24 | 0,18 | 0,24 | 0,19 | 0,17 | 0,18 | 0,18 | 0,15 | 0,21 | 0,23 | 0,22 | 0,24 | 0,20 | 0,16 | 0,17 | 0,18 |  |  |  |  |  |  |  |  |  |  |
| 31 | *Urocleidoides paradoxus* MT556795 | **0,20** | **0,20** | **0,21** | 0,24 | 0,24 | 0,23 | 0,22 | 0,23 | 0,22 | 0,19 | 0,18 | 0,25 | 0,23 | 0,26 | 0,18 | 0,24 | 0,20 | 0,18 | 0,17 | 0,17 | 0,08 | 0,22 | 0,23 | 0,22 | 0,23 | 0,20 | 0,18 | 0,19 | 0,18 | 0,11 |  |  |  |  |  |  |  |  |  |
| 32 | *Urocleidoides sinus* MT556799 | **0,21** | **0,22** | **0,22** | 0,23 | 0,22 | 0,23 | 0,23 | 0,25 | 0,24 | 0,18 | 0,17 | 0,23 | 0,25 | 0,25 | 0,19 | 0,24 | 0,21 | 0,19 | 0,18 | 0,18 | 0,16 | 0,21 | 0,24 | 0,22 | 0,23 | 0,20 | 0,18 | 0,18 | 0,18 | 0,10 | 0,11 |  |  |  |  |  |  |  |  |
| 33 | *Urocleidoides tenuis* MT556797 | **0,19** | **0,20** | **0,20** | 0,24 | 0,23 | 0,22 | 0,21 | 0,21 | 0,21 | 0,22 | 0,21 | 0,24 | 0,26 | 0,26 | 0,19 | 0,25 | 0,20 | 0,17 | 0,22 | 0,24 | 0,25 | 0,21 | 0,21 | 0,20 | 0,23 | 0,20 | 0,17 | 0,13 | 0,06 | 0,19 | 0,18 | 0,19 |  |  |  |  |  |  |  |
| 34 | *Urocleidoides triangulus* PQ530282 | **0,19** | **0,19** | **0,19** | 0,32 | 0,33 | 0,31 | 0,28 | 0,30 | 0,27 | 0,26 | 0,24 | 0,31 | 0,25 | 0,39 | 0,23 | 0,30 | 0,27 | 0,23 | 0,33 | 0,34 | 0,25 | 0,26 | 0,29 | 0,27 | 0,28 | 0,26 | 0,19 | 0,16 | 0,17 | 0,22 | 0,23 | 0,25 | 0,18 |  |  |  |  |  |  |
| 35 | *Urocleidoides saghirus* PQ530283 | **0,21** | **0,20** | **0,20** | 0,34 | 0,31 | 0,33 | 0,31 | 0,33 | 0,31 | 0,25 | 0,24 | 0,31 | 0,24 | 0,33 | 0,22 | 0,27 | 0,25 | 0,23 | 0,30 | 0,30 | 0,25 | 0,26 | 0,31 | 0,27 | 0,27 | 0,24 | 0,21 | 0,17 | 0,19 | 0,24 | 0,24 | 0,25 | 0,20 | 0,15 |  |  |  |  |  |
| 36 | *Urocleidoides taquariensis* PQ530284 | **0,17** | **0,17** | **0,17** | 0,22 | 0,22 | 0,20 | 0,19 | 0,20 | 0,20 | 0,19 | 0,18 | 0,21 | 0,23 | 0,25 | 0,17 | 0,24 | 0,19 | 0,16 | 0,22 | 0,23 | 0,24 | 0,18 | 0,19 | 0,19 | 0,22 | 0,19 | 0,15 | 0,11 | 0,11 | 0,17 | 0,17 | 0,18 | 0,11 | 0,09 | 0,15 |  |  |  |  |
| 37 | *Pseudorhabdosynochus lantauensis* AY553624 | **0,44** | **0,44** | **0,43** | 0,32 | 0,32 | 0,31 | 0,31 | 0,31 | 0,31 | 0,34 | 0,33 | 0,34 | 0,45 | 0,33 | 0,33 | 0,34 | 0,36 | 0,34 | 0,33 | 0,34 | 0,46 | 0,30 | 0,32 | 0,31 | 0,36 | 0,34 | 0,34 | 0,36 | 0,34 | 0,34 | 0,32 | 0,34 | 0,33 | 0,48 | 0,47 | 0,34 |  |  |  |
| 38 | *Pseudorhabdosynochus epinepheli* AY553622 | **0,44** | **0,44** | **0,43** | 0,31 | 0,32 | 0,30 | 0,30 | 0,31 | 0,30 | 0,35 | 0,34 | 0,33 | 0,44 | 0,33 | 0,32 | 0,34 | 0,35 | 0,34 | 0,32 | 0,33 | 0,44 | 0,30 | 0,31 | 0,31 | 0,36 | 0,34 | 0,34 | 0,35 | 0,33 | 0,33 | 0,32 | 0,33 | 0,33 | 0,49 | 0,45 | 0,34 | 0,12 |  |  |
| 39 | *Murraytrema pricei* DQ157672 | **0,41** | **0,41** | **0,40** | 0,35 | 0,35 | 0,32 | 0,32 | 0,31 | 0,33 | 0,35 | 0,34 | 0,34 | 0,46 | 0,37 | 0,34 | 0,32 | 0,35 | 0,34 | 0,33 | 0,33 | 0,45 | 0,33 | 0,33 | 0,32 | 0,36 | 0,35 | 0,33 | 0,35 | 0,34 | 0,33 | 0,32 | 0,34 | 0,34 | 0,47 | 0,46 | 0,33 | 0,26 | 0,26 |  |
