# Supplementary material for: Phylogenetic Placement of Whittingtonocotyle Neto, Rodrigues & Domingues, 2015 (Monopisthocotyla: Dactylogyridae) Inferred from the First Molecular Data of Both Described Species
Source: Acta Parasitol. 2026 Mar 9;71(2):59. doi: 10.1007/s11686-026-01239-8 (PMC12971823; doi:10.1007/s11686-026-01239-8)
Supplement: Supplementary file 3 — Supplementary Material 3 [file 11686_2026_1239_MOESM3_ESM.docx]

|  |  | 1 | 2 | 3 | 4 | 5 | 6 | 7 | 8 | 9 | 10 | 11 | 12 | 13 | 14 | 15 | **16** | **17** | 18 | 19 | 20 | 21 | 22 | 23 | 24 | 25 | 26 | 27 | 28 | 29 | 30 | 31 |
| --- | --- | --- | --- | --- | --- | --- | --- | --- | --- | --- | --- | --- | --- | --- | --- | --- | --- | --- | --- | --- | --- | --- | --- | --- | --- | --- | --- | --- | --- | --- | --- | --- |
| 1 | *Acanthocotyle gurgesiella* KY379331 |  |  |  |  |  |  |  |  |  |  |  |  |  |  |  |  |  |  |  |  |  |  |  |  |  |  |  |  |  |  |  |
| 2 | *Urocleidoides cultellus* MF939848 | 0,32 |  |  |  |  |  |  |  |  |  |  |  |  |  |  |  |  |  |  |  |  |  |  |  |  |  |  |  |  |  |  |
| 3 | *Urocleidoides gymnotus* OR270814 | 0,30 | 0,20 |  |  |  |  |  |  |  |  |  |  |  |  |  |  |  |  |  |  |  |  |  |  |  |  |  |  |  |  |  |
| 4 | *Urocleidoides carapus* OR270816 | 0,32 | 0,18 | 0,18 |  |  |  |  |  |  |  |  |  |  |  |  |  |  |  |  |  |  |  |  |  |  |  |  |  |  |  |  |
| 5 | *Urocleidoides uncinus* MT594473 | 0,29 | 0,26 | 0,26 | 0,24 |  |  |  |  |  |  |  |  |  |  |  |  |  |  |  |  |  |  |  |  |  |  |  |  |  |  |  |
| 6 | *Urocleidoides tenuis* MT594475 | 0,31 | 0,28 | 0,29 | 0,28 | 0,27 |  |  |  |  |  |  |  |  |  |  |  |  |  |  |  |  |  |  |  |  |  |  |  |  |  |  |
| 7 | *Urocleidoides nataliapasternakae* OR270823 | 0,31 | 0,27 | 0,25 | 0,26 | 0,23 | 0,27 |  |  |  |  |  |  |  |  |  |  |  |  |  |  |  |  |  |  |  |  |  |  |  |  |  |
| 8 | *Urocleidoides digitabulum* MT594400 | 0,33 | 0,31 | 0,31 | 0,30 | 0,31 | 0,25 | 0,28 |  |  |  |  |  |  |  |  |  |  |  |  |  |  |  |  |  |  |  |  |  |  |  |  |
| 9 | *Rhinoxenus paranaensis* OR064770 | 0,29 | 0,28 | 0,28 | 0,27 | 0,23 | 0,26 | 0,26 | 0,27 |  |  |  |  |  |  |  |  |  |  |  |  |  |  |  |  |  |  |  |  |  |  |  |
| 10 | *Urocleidoides taquariensis* PQ553541 | 0,29 | 0,28 | 0,29 | 0,27 | 0,22 | 0,25 | 0,23 | 0,25 | 0,22 |  |  |  |  |  |  |  |  |  |  |  |  |  |  |  |  |  |  |  |  |  |  |
| 11 | *Urocleidoides triangulus* PQ553670 | 0,29 | 0,30 | 0,27 | 0,29 | 0,26 | 0,24 | 0,24 | 0,23 | 0,25 | 0,17 |  |  |  |  |  |  |  |  |  |  |  |  |  |  |  |  |  |  |  |  |  |
| 12 | *Urocleidoides strombicirrus* MF939876 | 0,30 | 0,28 | 0,27 | 0,26 | 0,27 | 0,26 | 0,28 | 0,26 | 0,25 | 0,22 | 0,25 |  |  |  |  |  |  |  |  |  |  |  |  |  |  |  |  |  |  |  |  |
| 13 | *Urocleidoides saghirus* PQ553540 | 0,30 | 0,25 | 0,28 | 0,25 | 0,23 | 0,24 | 0,27 | 0,26 | 0,22 | 0,19 | 0,20 | 0,26 |  |  |  |  |  |  |  |  |  |  |  |  |  |  |  |  |  |  |  |
| 14 | *Jainus piava* OQ833543 | 0,26 | 0,25 | 0,26 | 0,27 | 0,26 | 0,26 | 0,26 | 0,23 | 0,20 | 0,19 | 0,23 | 0,22 | 0,21 |  |  |  |  |  |  |  |  |  |  |  |  |  |  |  |  |  |  |
| 15 | *Jainus beccus* OQ833545 | 0,26 | 0,22 | 0,25 | 0,25 | 0,25 | 0,24 | 0,26 | 0,25 | 0,21 | 0,20 | 0,20 | 0,22 | 0,19 | 0,14 |  |  |  |  |  |  |  |  |  |  |  |  |  |  |  |  |  |
| **16** | ***Whittingtonocotyle jeju* PZ044783** | **0,30** | **0,29** | **0,24** | **0,24** | **0,24** | **0,25** | **0,25** | **0,26** | **0,21** | **0,19** | **0,20** | **0,23** | **0,22** | **0,23** | **0,21** |  |  |  |  |  |  |  |  |  |  |  |  |  |  |  |  |
| **17** | ***Whittingtonocotyle caetei* PZ044784** | **0,30** | **0,29** | **0,24** | **0,25** | **0,24** | **0,26** | **0,25** | **0,26** | **0,21** | **0,19** | **0,19** | **0,23** | **0,22** | **0,22** | **0,21** | **0,01** |  |  |  |  |  |  |  |  |  |  |  |  |  |  |  |
| 18 | *Urocleidoides vanini* OR285309 | 0,30 | 0,29 | 0,29 | 0,28 | 0,28 | 0,26 | 0,28 | 0,27 | 0,24 | 0,22 | 0,19 | 0,26 | 0,21 | 0,23 | 0,23 | **0,22** | **0,22** |  |  |  |  |  |  |  |  |  |  |  |  |  |  |
| 19 | *Urocleidoides macrosoma* OR270815 | 0,30 | 0,26 | 0,27 | 0,25 | 0,25 | 0,25 | 0,25 | 0,25 | 0,23 | 0,18 | 0,20 | 0,22 | 0,23 | 0,22 | 0,21 | **0,18** | **0,18** | 0,23 |  |  |  |  |  |  |  |  |  |  |  |  |  |
| 20 | *Urocleidoides naris* OR285308 | 0,29 | 0,29 | 0,27 | 0,26 | 0,25 | 0,26 | 0,25 | 0,24 | 0,24 | 0,23 | 0,21 | 0,23 | 0,22 | 0,23 | 0,22 | **0,18** | **0,18** | 0,20 | 0,17 |  |  |  |  |  |  |  |  |  |  |  |  |
| 21 | *Urocleidoides malabaricusi* KT625589 | 0,30 | 0,28 | 0,26 | 0,26 | 0,26 | 0,24 | 0,25 | 0,25 | 0,23 | 0,21 | 0,21 | 0,24 | 0,24 | 0,21 | 0,22 | **0,18** | **0,18** | 0,20 | 0,17 | 0,15 |  |  |  |  |  |  |  |  |  |  |  |
| 22 | *Diaphorocleidus neotropicalis* MZ408254 | 0,33 | 0,28 | 0,28 | 0,26 | 0,27 | 0,27 | 0,29 | 0,27 | 0,25 | 0,23 | 0,24 | 0,19 | 0,26 | 0,23 | 0,24 | **0,22** | **0,23** | 0,23 | 0,22 | 0,22 | 0,23 |  |  |  |  |  |  |  |  |  |  |
| 23 | *Urocleidoides malabaricusi* KT625588 | 0,32 | 0,29 | 0,28 | 0,29 | 0,25 | 0,23 | 0,23 | 0,25 | 0,23 | 0,21 | 0,20 | 0,27 | 0,23 | 0,24 | 0,24 | **0,20** | **0,20** | 0,18 | 0,20 | 0,20 | 0,20 | 0,26 |  |  |  |  |  |  |  |  |  |
| 24 | *Urocleidoides malabaricusi* KT625587 | 0,32 | 0,28 | 0,28 | 0,28 | 0,25 | 0,23 | 0,27 | 0,24 | 0,23 | 0,21 | 0,22 | 0,26 | 0,23 | 0,23 | 0,25 | **0,20** | **0,20** | 0,20 | 0,20 | 0,18 | 0,17 | 0,23 | 0,16 |  |  |  |  |  |  |  |  |
| 25 | *Diaphorocleidus magnus* MZ408253 | 0,28 | 0,26 | 0,26 | 0,26 | 0,24 | 0,25 | 0,26 | 0,26 | 0,21 | 0,19 | 0,21 | 0,20 | 0,22 | 0,20 | 0,21 | **0,19** | **0,19** | 0,23 | 0,20 | 0,21 | 0,23 | 0,19 | 0,25 | 0,24 |  |  |  |  |  |  |  |
| 26 | *Diaphorocleidus petrosusi* MF939866 | 0,24 | 0,26 | 0,25 | 0,26 | 0,23 | 0,23 | 0,26 | 0,23 | 0,22 | 0,19 | 0,20 | 0,19 | 0,18 | 0,19 | 0,21 | **0,20** | **0,21** | 0,21 | 0,20 | 0,19 | 0,19 | 0,17 | 0,19 | 0,20 | 0,17 |  |  |  |  |  |  |
| 27 | *Urocleidoides sinus* MT594474 | 0,27 | 0,29 | 0,29 | 0,27 | 0,25 | 0,26 | 0,27 | 0,22 | 0,24 | 0,20 | 0,21 | 0,23 | 0,24 | 0,23 | 0,21 | **0,22** | **0,22** | 0,24 | 0,21 | 0,21 | 0,19 | 0,24 | 0,25 | 0,23 | 0,23 | 0,23 |  |  |  |  |  |
| 28 | *Jainus radixelongatus* OQ833544 | 0,25 | 0,24 | 0,24 | 0,22 | 0,24 | 0,22 | 0,20 | 0,20 | 0,21 | 0,18 | 0,20 | 0,19 | 0,17 | 0,20 | 0,18 | **0,19** | **0,19** | 0,23 | 0,17 | 0,18 | 0,19 | 0,19 | 0,19 | 0,16 | 0,20 | 0,19 | 0,16 |  |  |  |  |
| 29 | *Urocleidoides itabocaensis* PP118263 | 0,31 | 0,28 | 0,30 | 0,26 | 0,24 | 0,27 | 0,26 | 0,23 | 0,21 | 0,20 | 0,19 | 0,23 | 0,23 | 0,23 | 0,23 | **0,20** | **0,21** | 0,22 | 0,21 | 0,22 | 0,21 | 0,23 | 0,21 | 0,21 | 0,21 | 0,21 | 0,21 | 0,19 |  |  |  |
| 30 | *Urocleidoides omphalocleithrum* PP106159 | 0,31 | 0,28 | 0,29 | 0,28 | 0,23 | 0,26 | 0,26 | 0,22 | 0,23 | 0,18 | 0,21 | 0,24 | 0,23 | 0,20 | 0,19 | **0,20** | **0,19** | 0,23 | 0,22 | 0,22 | 0,19 | 0,23 | 0,21 | 0,19 | 0,22 | 0,22 | 0,21 | 0,19 | 0,15 |  |  |
| 31 | *Urocleidoides curvocuspidis* OR582424 | 0,28 | 0,27 | 0,26 | 0,26 | 0,24 | 0,23 | 0,25 | 0,23 | 0,23 | 0,20 | 0,20 | 0,23 | 0,21 | 0,21 | 0,19 | **0,18** | **0,18** | 0,25 | 0,20 | 0,20 | 0,20 | 0,21 | 0,21 | 0,21 | 0,20 | 0,16 | 0,19 | 0,16 | 0,18 | 0,14 |  |
